# Cardiovascular Mortality in Type 2 Diabetes Patients with Incident Exposure to Insulin Glargine

**DOI:** 10.1155/2015/962346

**Published:** 2015-06-14

**Authors:** Sorin Ioacara, Cristian Guja, Aura Reghina, Sorina Martin, Anca Sirbu, Simona Fica

**Affiliations:** ^1^“Elias” Emergency University Hospital, Marasti Boulevard 17, 011461 Bucharest, Romania; ^2^“Carol Davila” University of Medicine and Pharmacy, Eroii Sanitari 8, 050474 Bucharest, Romania; ^3^“Prof. NC Paulescu” National Institute of Diabetes, Nutrition and Metabolic Diseases, Ion Movila 5-7, 020475 Bucharest, Romania; ^4^“Victor Babes” National Research and Development Institute of Pathology and Biomedical Sciences, Splaiul Independentei 99-101, 050096 Bucharest, Romania

## Abstract

The study investigated the impact of insulin glargine exposure on cardiovascular mortality in type 2 diabetes patients with incident insulin initiation. All consecutive diabetes patients aged >40 years were screened at their first diabetes outpatient visit between 01/01/2001 and 12/31/2008 (*n* = 79869). Exclusion criteria restricted the cohort to 4990 incident insulin users, aged 40–79 years, who were followed up for death until 12/31/2011. Baseline was defined 6 months after insulin initiation. Adjusted time-dependent competing risk regression analysis was performed. Mean baseline age was 62 ± 9 years, with mean follow-up of 4.7 ± 1.9 years. During 23179 person-years of exposure time, there were 887 deaths (521 cardiovascular). Glargine cumulative time exposure significantly lowered overall cardiovascular, subhazard ratio (SHR) 0.963 (CI 95% 0.944–0.981, *p* < 0.001), and myocardial infarction mortality, SHR 0.945 (CI 95% 0.899–0.994, *p* = 0.028), but not stroke mortality. Glargine cumulative dose exposure (10,000 IU increments) significantly lowered cardiovascular mortality, SHR 0.977 (CI 95% 0.960–0.993, *p* = 0.006), but not for myocardial infarction and stroke. Both cumulative dose and time exposure to insulin glargine were associated with lower cardiovascular mortality. The effect was mostly driven by myocardial infarction end point, supporting the concept of macrovascular benefit for basal analogue insulin use in type 2 diabetes.

## 1. Introduction

Several studies indicated that type 2 diabetes (T2D) significantly reduces life expectancy [1, 2], mainly as a consequence of increased rates of cardiovascular events [3]. Thus, it was repeatedly shown that cardiovascular disease is the main cause of death among patients with diabetes mellitus, leading to a threefold increase in cardiovascular mortality [4]. However, there is an open debate if intensive diabetes control has an impact on cardiovascular mortality in T2D patients [5].

Despite being used for almost 100 years, exogenous insulin is still surrounded by controversy regarding its effect on atherosclerosis risk and consequent CV events [6]. Diabetes being a progressive disease, the percentage of T2D patients using insulin increases in parallel with the duration of the disease [7, 8]. Insulin glargine is a basal insulin analog widely used in the management of both type 1 and type 2 diabetes. Recently, the ORIGIN trial showed that insulin glargine has no effect on cardiovascular events rate in subjects with early dysglycemia [9]. However, there are scarce data regarding its effect on cardiovascular mortality in subjects with long standing T2D.

The aim of the study was to investigate the impact of insulin glargine exposure on cardiovascular mortality in T2D patients with incident insulin initiation.

## 2. Materials and Methods

All consecutive diabetes patients (*n* = 79869) attending their regular consultation in “Ion Pavel” and “Nicolae Malaxa” outpatient clinics, Bucharest, Romania, were screened between 2001 and 2008. Details about cohort inception and some results on cancer mortality were published elsewhere [10]. For the purpose of this observational study we focused on T2D patients treated only with oral antidiabetic drugs (OADs) at screening, who maintained OADs only for at least six months. They were all initiated on insulin before 2011, with at least six months of continuous insulin exposure. These patients were considered as coming under observation with the occasion of their first diabetes prescription (screening) and coming at risk (cohort inception, meaning real start of follow-up) at six months after insulin initiation. We excluded patients aged less than 40 years or above 80 years for a greater generalization of study results. The final cohort retained 4990 subjects, respecting all the inclusion and meeting none of the exclusion criteria. All patients were then followed up for general and cause-specific mortality until 31 December 2011, by cross-linking with National Institute of Statistics database. Mortality data was based on death certificates, using the International Statistical Classification of Diseases and Related Health Problems 10th Revision (ICD-10; http://www.who.int/classifications/icd/en/). The primary outcome was cardiovascular mortality, ICD-10 codes I00–I99. Secondary outcomes were defined as fatal myocardial infarction (ICD-10 codes I21–I23) and fatal stroke (ICD-10 codes I61–I64). Deaths from other causes were defined as competing events.

All diabetes prescription information, including the doses used for each medication, was available from screening until death or 31 December 2008, whichever came first. All data in this respect from 2009 onward was used as last observation carried forward (LOCF). This minimizes the reverse causation, while adding some acceptable treatment misclassification at the end of follow-up in some patients. Both insulin and oral medications were included in the analyses. Other confounders were gender, age, observation time until first insulin exposure, and a composite variable expressing treatment intensity level (TIL) at the moment of insulin initiation. As a proxy for disease severity, TIL was constructed as the sum of every oral medication dose divided by the standard mean dose of that particular medication as explained elsewhere [10, 11].

Statistical analysis was performed with (non)parametric test deployed as appropriate. Time and dose exposure to various diabetes treatments were modeled as time-dependent variables, with daily updates of the system. The impact of glargine exposure on cardiovascular mortality was assessed by competing risk analysis with time-dependent variables, which is the best approach in the presence of significant competing events [12]. All cumulative dose risks were expressed for a 10,000 mg (oral) or U (insulin) increment. Detemir insulin and GLP1 agonists were excluded from the analysis as they only become available towards the end of the study period. The inherent “frailty” of the data resulting from the lack of randomization was addressed by the concomitant use of cumulative time/dose and binary ever/never exposed variables for all treatment modalities [13]. A standard sensitivity analysis completed the data mining, including “fixed-cohort,” restricted cumulative dose until one year prior to event or end of follow-up, propensity score, and standard Cox regression analysis (time-dependent). All statistical analyses were performed using STATA 13 (http://www.stata.com/).

The study was approved by the local ethics committee and performed according to the Helsinki Declaration.

## 3. Results

### 3.1. As-Treated Analysis

There were 4990 cases (58.4% females) of incident insulin users, mean age at inception 62.1 ± 9.3 years (62.8 ± 9.3 years for females versus 61.2 ± 9.2 years for males, *p* < 0.001), with a mean follow-up time from screening to insulin initiation (prestudy) follow-up time of 4.45 ± 1.91 years (4.51 ± 1.90 years for females versus 4.37 ± 1.93 years for males, *p* = 0.015). Patients were exposed to risk (insulin) for a mean of 4.6 ± 1.9 years (23179 person-years) of follow-up, starting at 6 months after insulin initiation. The mean insulin dose during follow-up was 36 ± 14 U, with no gender differences. Patients continued to take various oral medications at cohort inception (6 months following insulin initiation), that is, sulphonylurea (22.8%), metformin (23.7%), repaglinide (3.2%), pioglitazone (2.5%), and rosiglitazone (1.1%).

**Figure 1:**
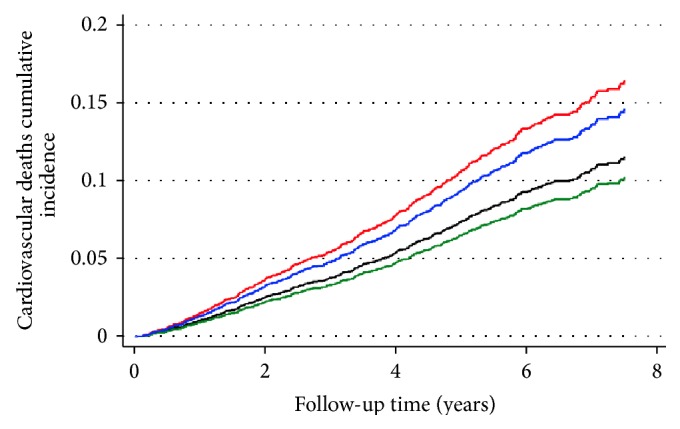
Cardiovascular deaths cumulative incidence functions. Green line: females exposed to glargine; black line: females unexposed to glargine; blue line: males exposed to glargine; red line: males unexposed to glargine.

There were 521 cardiovascular deaths (58.7% of total deaths), corresponding to a crude mortality rate of 22.5/1000 person-years. Cumulative time exposure to glargine had a SHR (subhazard ratios, similar to hazard ratios, HR, from the classic Cox regression) for total cardiovascular mortality of 0.963 (CI 95% 0.944–0.981, *p* < 0.001), while cumulative dose exposure had a SHR of 0.977 (CI 95% 0.960–0.993, *p* = 0.006). As regards the myocardial infarction, there were 152 deaths (6.56/1000 person-years' CMR), translating into a glargine cumulative time exposure SHR of 0.945 (CI 95% 0.899–0.994, *p* = 0.028) and cumulative dose exposure SHR of 0.961 (CI 95% 0.922–1.002, *p* = 0.064). Detailed information is given in Table 1. Similarly, there were 130 deaths from stroke (5.61/1000 person-years' CMR), with a glargine SHR for cumulative time of 0.973 (CI 95% 0.927–1.020, *p* = 0.257) and for cumulative dose of 0.974 (CI 95% 0.929–1.021, *p* = 0.266), as detailed in Table 2. Age was a significant risk factor for both myocardial infarction and stroke mortality (SHR 1.05, *p* < 0.001; and SHR 1.1, *p* < 0.001 resp.), while male gender was a significant risk factor for fatal myocardial infarction (SHR 2.1, *p* < 0.001) but not for stroke (SHR 1.2, *p* = 0.4) (Tables 1 and 2).  Figure 1 presents the cumulative incidence curves for cardiovascular mortality, using a 2 by 2 factorial split of data by gender and glargine exposure (ever versus never exposed).

### 3.2. Sensitivity Analysis

Limiting the cumulative exposure to that attained one year before death or end of original follow-up (tackling reverse causation) yielded similar results for both cumulative time SHR 0.940 (CI 95% 0.919–0.960, *p* < 0.001) and cumulative dose SHR 0.954 (CI 95% 0.934–0.974, *p* < 0.001). “Fixing” the diabetes treatment to the option received at cohort inception showed a glargine exposure associated HR (standard Cox regression) for cardiovascular mortality of 1.369 (CI 95% 0.809–2.316, *p* = 0.242). Propensity score analysis did not significantly alter any of the above risk estimates.

## 4. Discussion

In this study of 4990 incident insulin users suffering from type 2 diabetes, who were followed up for 23179 person-years, there was a statistical significant benefit from cumulative time exposure to insulin glargine for specific mortality due to cardiovascular diseases (SHR 0.963, CI 95% 0.944–0.981, *p* < 0.001). This benefit appears to be mostly driven by the myocardial infarction (SHR 0.945, CI 95% 0.899–0.994, *p* = 0.028), but not stroke mortality (SHR 0.973, CI 95% 0.927–1.020, *p* = 0.257). This finding is further supported by the glargine cumulative dose exposure analysis showing a protective effect on cardiovascular mortality (SHR 0.977, CI 95% 0.960–0.993, *p* = 0.006), with a borderline not significant result for myocardial infarction (SHR 0.961, CI 95% 0.922–1.002, *p* = 0.064), and clearly no impact on fatal stroke (SHR 0.974, CI 95% 0.929–1.021, *p* = 0.266). This is consistent with the general finding of better metabolic control having a benefit on fatal myocardial infarction, but not stroke, and blood pressure control lowering the stroke risk, but not myocardial infarction [14, 15]. Although no metabolic control marker was available in this study, it is very much more likely that glargine exposure would impact the metabolic control rather than blood pressure.

Previous reports indicated no effect of basal insulin initiation on fatal and nonfatal cardiovascular events in patients with prediabetes or recent onset T2D [9]. More recently, insulin treatment initiation in T2D patients treated with metformin alone for a median of 14 months led to an increase of a composite end point including cardiovascular events and all-cause mortality but had no effect on cardiovascular mortality alone [16]. The contrasting results of our study, with cardiovascular mortality benefits for insulin initiation, could be explained by addressing the same relevant question in significantly different population. We explored the very much more common situation of insulin initiation in long standing T2D patients, with secondary failure of OADs only treatment. With increasing life expectancy in this population, we expect this question (and its answer) to become more and more relevant in the future [17, 18].

As a strong point, this study manages to enroll a significant amount of rather homogenous patients, very likely to represent the “real life” type 2 diabetes patients, treated with OGLD only, who come to the point of insulin requirement. Differences in disease progression at insulin initiation were dealt with by the “treatment intensity level” composite index, which was constructed as a function of number and dose of OGLD at the moment of insulin start-up. Exposure to all insulin and oral medication modalities was recorded as time-dependent variable, ensuring the best estimate of their impact on mortality. Reverse causation introduced by a rapidly deteriorating cardiovascular disease resulting in final life-days switching on insulin was prevented by enforcing at least 6 months of “immortality” following insulin initiation. As stated above in study design, the follow-up starts after this initial observational time has successfully passed, avoiding the inherent immortal time bias. The age and gender were both available for analysis and proved to be important confounders to adjust for. Sensitivity analysis was performed in order to ensure the best confidence in tackling the reverse causation surrounding either the end of follow-up (exposure restricted to one year prior to death or end of follow-up) or its beginning (“fixed-cohort analysis”).

As a week point, many other important cardiovascular risk factors, including but not limiting to smoking, body mass index, physical activity, or lipid profile, were not available in this study. However, there are very few doctors who would decide to use a specific type of insulin based on smoking or lipid status, resulting, in our particular case, in a lower impact on results of this unmeasured confounder. One can also comment that the risk of a major cardiovascular event is so high in T2D patients requiring insulin treatment and that the additional risk brought about by the allocation bias is very much diluted. Glycated haemoglobin was not available, but there was no local official or unofficial recommendation towards use of a specific threshold for choosing between the various insulin options. As regards the particular case of insulin glargine, there is a high chance that doctors used it both in severe insulin deficient patients as part of the basal bolus regime and in less insulin deficient cases as basal only regime. This spread range of glargine usage is likely to also be present in patients with various degrees of cardiovascular involvement. Although the blood pressure and cardiovascular status of these patients were not available, there was no local indication or contraindication for any insulin option in relation to this unmeasured confounder. One can speculate that for any particular age group (available here as a confounder) diabetes patients with a less cardiovascular burden would be more likely to receive the basal bolus regime as compared with those with a more advanced cardiovascular disease who would be more likely to receive the basal only regime. As stated above, in the particular case of insulin glargine, that would probably not translate in significant differences in exposure due to its similar use in both regimes. The local clinical practice included routine cardiovascular risk factors evaluation and careful treatment according to generally agreed guidelines. The results of these cardiovascular risk assessments were not available in our study, but it was shown that regular treatment of those risk factors (done at the population level) ensures similar outcomes as compared with extensive cardiovascular disease assessment, and therefore current guidelines do not recommend cardiovascular screening as long as cardiovascular risk factors are treated. Furthermore, it was demonstrated that the residual confounding resulting from the lack of randomisation in observational studies can be successfully accounted for by the concomitant use of time-dependent ever exposed and cumulative exposure variables [13]. The follow-up time is rather short for a cardiovascular mortality end point. Results can still be interpreted as a significant and beneficial impact of glargine exposure on cardiovascular mortality that is readily evident after around four years of follow-up (short term mortality). Using cardiovascular mortality instead of cardiovascular incidence and data from the death certificates are important drawbacks. Still, cardiovascular mortality has very high rates in the study population, which ensures a very good correlation between cardiovascular disease incidence and mortality. While an absolute error of measurement is clearly inevitable, it is likely that it applies similarly to all insulin modalities evaluated, without a significant bias resulting from favouring a specific insulin. Although all measures were taken to have the best possible evaluation of the independent effect of a particular insulin type on cardiovascular mortality, in reality this effect was calculated against the “medium” exposure to all other types of insulin due to the fact that all patients received insulin in various forms. So, the results of any insulin type can be interpreted as adjusted for any past or concomitant other insulin options.

## 5. Conclusion

Exposure to insulin glargine in long standing T2D patients, who reach the point of adding or switching to insulin after a certain (usually long) time on OADs alone, is safe and maybe even beneficial in terms of major fatal cardiovascular events. Therefore, we recommend basal insulin use as a safe method in achieving the individualised target of metabolic control in type 2 diabetes patients.

## Figures and Tables

**Table 1:** Competing risk analysis for myocardial infarction mortality.

Variables	Cumulative time		Cumulative dose	
	SHR (CI 95%)^a^	*p*	SHR (CI 95%)	*p*
Deaths (*n*)	152	—	152	—
Age at inception	1.054 (1.035–1.074)	<0.001	1.053 (1.034–1.072)	<0.001
Male gender	2.149 (1.559–2.964)	<0.001	2.091 (1.513–2.889)	<0.001
TIL^b^	0.960 (0.744–1.239)	0.753	0.979 (0.760–1.262)	0.873
Screening time^c^	0.928 (0.849–1.014)	0.100	0.935 (0.855–1.022)	0.138
Glargine	0.945 (0.899–0.994)	0.028	0.961 (0.922–1.002)	0.064
Basal human insulin	0.958 (0.919–0.999)	0.044	0.975 (0.920–1.034)	0.400
Regular insulin	1.017 (0.990–1.043)	0.217	0.990 (0.975–1.005)	0.199
Rapid-acting analogs	1.002 (0.943–1.065)	0.943	0.988 (0.941–1.037)	0.632
Premixed human insulin	0.960 (0.939–0.981)	<0.001	0.972 (0.954–0.990)	0.002
Premixed analogue insulin	0.956 (0.927–0.987)	0.005	0.983 (0.961–1.006)	0.143
Metformin	0.979 (0.942–1.018)	0.291	0.999 (0.999–1.000)	0.540
Glimepiride	1.013 (0.952–1.078)	0.679	1.060 (0.743–1.514)	0.748
Gliclazide	0.995 (0.922–1.074)	0.907	0.991 (0.965–1.019)	0.536
Glipizide	1.007 (0.924–1.097)	0.878	0.996 (0.873–1.136)	0.947
Glibenclamide	0.984 (0.931–1.040)	0.561	0.979 (0.832–1.153)	0.801
Repaglinide	0.894 (0.770–1.038)	0.142	0.296 (0.079–1.103)	0.070
Pioglitazone	0.992 (0.921–1.068)	0.832	1.002 (0.955–1.051)	0.943
Rosiglitazone	0.887 (0.722–1.091)	0.256	0.684 (0.336–1.390)	0.294

^a^SHR: subhazard ratios, similar to hazard ratios (HR) from the classic Cox regression.

^b^Treatment intensity level (see Section 2).

^c^Time between screening and date of insulin initiation (years).

**Table 2:** Competing risk analysis for stroke mortality.

Variables	Cumulative time		Cumulative dose	
	SHR (CI 95%)^a^	*p*	SHR (CI 95%)	*p*
Deaths (*n*)	130	—	130	—
Age at inception	1.102 (1.079–1.125)	<0.001	1.102 (1.079–1.126)	<0.001
Male gender	1.168 (0.820–1.662)	0.390	1.163 (0.818–1.655)	0.400
TIL^b^	1.118 (0.838–1.491)	0.450	1.140 (0.855–1.521)	0.372
Screening time^c^	0.892 (0.816–0.975)	0.012	0.897 (0.820–0.980)	0.016
Glargine	0.973 (0.927–1.020)	0.257	0.974 (0.929–1.021)	0.266
Basal human insulin	0.852 (0.747–0.973)	0.018	0.838 (0.727–0.967)	0.016
Regular insulin	0.994 (0.962–1.027)	0.724	0.996 (0.976–1.015)	0.668
Rapid-acting analogs	0.971 (0.875–1.076)	0.571	0.941 (0.868–1.020)	0.139
Premixed human insulin	0.990 (0.969–1.012)	0.372	1.002 (0.990–1.014)	0.729
Premixed analogue insulin	0.957 (0.931–0.984)	0.002	0.981 (0.957–1.006)	0.133
Metformin	0.958 (0.904–1.015)	0.149	0.999 (0.999–1.000)	0.344
Glimepiride	0.972 (0.889–1.062)	0.526	0.948 (0.546–1.649)	0.851
Gliclazide	0.854 (0.734–0.993)	0.040	0.947 (0.893–1.004)	0.068
Glipizide	0.895 (0.760–1.053)	0.181	0.875 (0.706–1.084)	0.221
Glibenclamide	0.907 (0.821–1.001)	0.053	0.818 (0.624–1.072)	0.145
Repaglinide	0.927 (0.836–1.028)	0.150	0.791 (0.494–1.265)	0.327
Pioglitazone	0.936 (0.789–1.110)	0.446	0.978 (0.889–1.076)	0.652
Rosiglitazone	0.866 (0.756–0.993)	0.039	0.557 (0.347–0.896)	0.652

^a^SHR: subhazard ratios, similar to hazard ratios (HR) from the classic Cox regression.

^b^Treatment intensity level (see Section 2).

^c^Time between screening and date of insulin initiation (years).

## References

[1] Roglic G., Unwin N. (2010). Mortality attributable to diabetes: estimates for the year 2010. *Diabetes Research and Clinical Practice*.

[2] Seshasai S. R. K., Kaptoge S., Thompson A. (2011). Diabetes mellitus, fasting glucose, and risk of cause-specific death. *The New England Journal of Medicine*.

[3] Barnett K. N., Ogston S. A., McMurdo M. E. T., Morris A. D., Evans J. M. M. (2010). A 12-year follow-up study of all-cause and cardiovascular mortality among 10,532 people newly diagnosed with type 2 diabetes in Tayside, Scotland. *Diabetic Medicine*.

[4] Taylor K. S., Heneghan C. J., Farmer A. J. (2013). All-cause and cardiovascular mortality in middle-aged people with type 2 diabetes compared with people without diabetes in a large U.K. primary care database. *Diabetes Care*.

[5] Konig M., Lamos E. M., Stein S. A., Davis S. N. (2013). An insight into the recent diabetes trials: what is the best approach to prevent macrovascular and microvascular complications?. *Current Diabetes Reviews*.

[6] Nandish S., Bailon O., Wyatt J. (2011). Vasculotoxic effects of insulin and its role in atherosclerosis: what is the evidence?. *Current Atherosclerosis Reports*.

[7] Turner R. C., Cull C. A., Frighi V., Holman R. R. (1999). Glycemic control with diet, sulfonylurea, metformin, or insulin in patients with type 2 diabetes mellitus: progressive requirement for multiple therapies (UKPDS 49). UK Prospective Diabetes Study (UKPDS) Group. *The Journal of the American Medical Association*.

[8] Joseph J. J., Donner T. W. (2015). Long-term insulin glargine therapy in type 2 diabetes mellitus: a focus on cardiovascular outcomes. *Vascular Health and Risk Management*.

[9] The ORIGIN Trial Investigators, Gerstein H. C., Bosch J. (2012). Basal insulin and cardiovascular and other outcomes in dysglycemia. *The New England Journal of Medicine*.

[10] Ioacara S., Guja C., Ionescu-Tirgoviste C., Fica S., Roden M. (2014). Cancer specific mortality in insulin-treated type 2 diabetes patients. *PLoS ONE*.

[11] WHO Collaborating Centre for Drug Statistics Methodology (2012). *ATC/DDD Index*.

[12] Fine J. P., Gray R. J. (1999). A proportional hazards model for the subdistribution of a competing risk. *Journal of the American Statistical Association*.

[13] Colhoun H. M., Livingstone S. J., Looker H. C. (2012). Hospitalised hip fracture risk with rosiglitazone and pioglitazone use compared with other glucose-lowering drugs. *Diabetologia*.

[14] Gerstein H. C., Miller M. E., Genuth S. (2011). Long-term effects of intensive glucose lowering on cardiovascular outcomes. *The New England Journal of Medicine*.

[15] Reboldi G., Gentile G., Angeli F., Ambrosio G., Mancia G., Verdecchia P. (2011). Effects of intensive blood pressure reduction on myocardial infarction and stroke in diabetes: a meta-analysis in 73,913 patients. *Journal of Hypertension*.

[16] Roumie C. L., Greevy R. A., Grijalva C. G. (2014). Association between intensification of metformin treatment with insulin vs sulfonylureas and cardiovascular events and all-cause mortality among patients with diabetes. *The Journal of the American Medical Association*.

[17] Ioacara S., Guja C., Ionescu-Tirgoviste C. (2011). Improvements in life expectancy in adult type 2 diabetes patients in the last six decades. *Diabetes Research and Clinical Practice*.

[18] Ioacara S., Guja C., Fica S., Ionescu-Tirgoviste C. (2013). The dynamics of life expectancy over the last six decades in elderly people with diabetes. *Diabetes Research and Clinical Practice*.

